# Vocal Cord Paralysis after Tracheal Intubation: An Overview of the Etiology and Associated Risk Factors

**DOI:** 10.1055/s-0045-1808244

**Published:** 2025-07-07

**Authors:** Meerab Anwar, Komal Ashiq Hussain, Pervez Anwar

**Affiliations:** 1 Department of Biotechnology, Faculty of Sciences, University of Sialkot, Sialkot, Punjab, Pakistan; 2 Department of Biochemistry, Faculty of Sciences, University of Sialkot, Sialkot, Punjab, Pakistan

**Keywords:** vocal cord, etiology, idiopathic, intubation, comorbidities

## Abstract

**Introduction:**

Vocal cord paralysis (VCP) is a deprivation of motility and the dysfunction of the vocal cords due to a defect in the vagal nerve or recurrent laryngeal nerve (RLN). It also occurs due to mutilation in the cricoarytenoid joint or posterior commissure scarring after prolonged tracheal intubation. It is a disorder with an extensive range of etiologies reliant on its laterality pattern.

**Objective:**

To discuss the laterality pattern of VCP due to endotracheal intubation and its respective treatments, the associated etiologies, and the risk factors, to provide a new direction to physicians for its treatment and to avoid its occurrence.

**Data Synthesis:**

We conducted a peer review of many of the articles published to date on VCP. An analysis of 967 patients from 5 studies determined that unilateral VCP (UVCP) is three times more frequent than bilateral VCP (BVCP). Furthermore, we analyzed 2,232 patients from 6 different studies that concluded surgery was the most common cause, followed by neoplastic diseases or malignancies. Another important though highly uncommon etiology of VCP is endotracheal intubation; however, it is disturbing for doctors as endotracheal intubation is a common procedure for general anesthesia.

**Conclusion:**

A variety of factors may cause VCP, including age, comorbidities, body mass index BMI, the duration of the intubation, the handling of the apparatus, operative time, and tracheal tube sizes. Preventive measures should be prioritized to avoid severe consequences, and intubation must be performed carefully in elderly people and in subjects with lower BMIs. Knowledge of the risk factors will help physicians customize intubation procedures in the future.

## Introduction

The tissue folds on the inner side of the larynx are called *vocal cords* , which play a role in phonation and in air flow regulation into the lungs. 1 The deprivation of the typical motility and the dysfunction of the vocal cords due to a lesion is called *vocal cord paralysis* or (VCP) *vocal cord palsy* , which is a primary indication of numerous severe underlying ailments. 2 Vocal cord paralysis actually encompasses the defect in the vagal nerve or in one of its branches, called the *recurrent laryngeal nerve* (RLN), which innervates the muscle responsible for the movement of the vocal cords, and it must not confused with the condition in which vocal cord fixation occurs on an anatomic level. 3 In VCP, the vocal cords cannot properly close or open. The condition can either be congenital or acquired. 4 We herein review the types of VCP, its laterality rates, etiology, and the risk factors associated with VCP due to tracheal intubation, which is one of the underlying complications linked to this procedure. 5

## Literature Review and Discussion

### Types of Vocal Cord Paralysis

**Figure 1:**
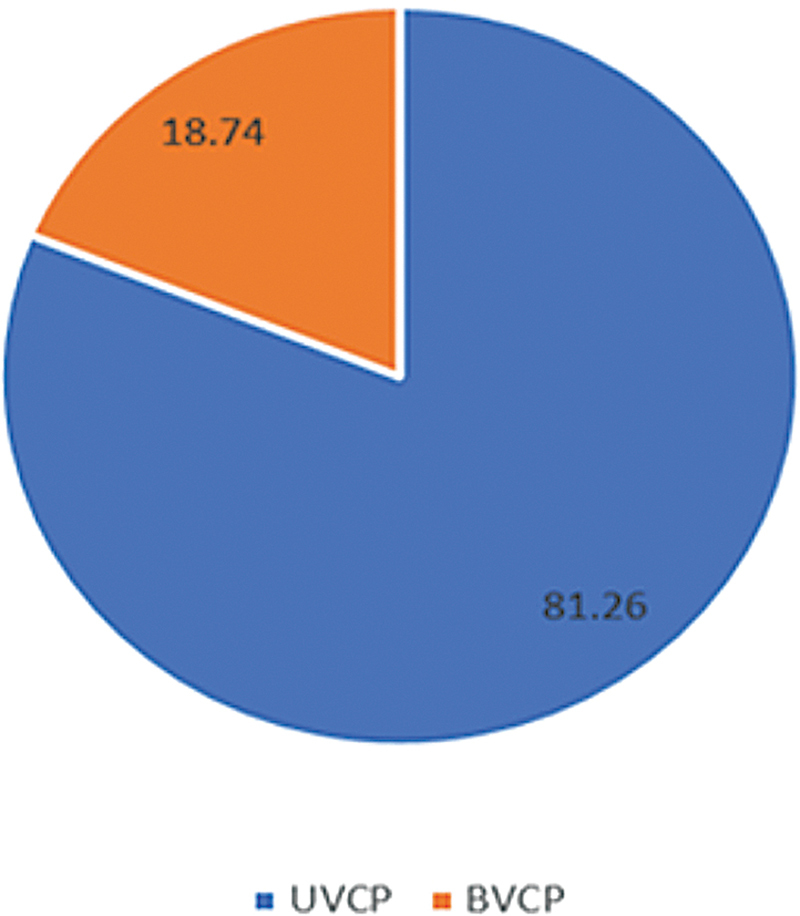
Pie chart illustrates the laterality pattern of vocal cord paralysis.

The two major classes of VCP are unilateral (UVCP) a bilateral (BVCP), and they differ in terms of the root of their symptoms, etiology, and pathologies ( Figure 1 ).

Bilateral VCP is rare disorder ( Table 1 ) of complete absence of mobility of both vocal cords. Regarding its causes, 59% of the cases of BVCP are congenital, and 41%, acquired. 6 Bilateral VCP is an immense challenge faced by pediatric otolaryngologists, as it is the second most common cause of neonatal stridor after laryngomalacia. 7 8 Its symptoms are dysphonia and aspiration that ultimately tracks breathing and speaking troubles, inspiratory dyspnea, respiratory distress, voice change, and swallowing glitches. 3 7 9 Although BVCP often causes severe effects, it can be reverted through various procedures dependent on the position of vocal folds, such as tracheostomy, laterofixation, reinnervation, endoscopic arytenoid abduction lateropexy (EAAL), and stem cell therapy. 10 11 12 13 14

**Table 1:** Meta-analysis of reports of UVCP and BVCP

Total of cases	UVCP	BVCP	Diagnostic techniques used	Study
**113**	74%	26%	Chest roentgenogram	Terris et al. 47 (1992)
**120**	93.3%	6.67%	Indirect laryngoscopy, laryngeal endoscopy	Gupta et al. 3 (2013)
**100**	86%	14%	Indirect laryngoscopy	Parnell and Brandenburg 48 (1970)
**181**	70%	30%	CBC, VDRL, radiology, endoscopy	Maisel and Ogura 49 (1974)
**453**	83%	17%	CT, MRI, CXR, EMG	Rosenthal et al. 15 (2007)

Abbreviations: BVCP, bilateral vocal cord paralysis; CT, computed tomography; CXR, chest X-ray; EMG, electromyography; MRI, magnetic resonance imaging; UVCP, unilateral vocal cord paralysis.

On the other hand, UVCP is extremely common in otolaryngology practice, and it is three times more frequent than BVCP. There is a consensus that iatrogenic injury is the most frequent cause of UVCP; however, regarding the cases related to surgery, thyroid surgeries are the leading cause. 15 Unilateral VCP is additionally categorized into left and right vocal cord paralysis. Left RLN paralysis is two times more common than on the right side, because, within the mediastinum, the lengthier nerve passage generates more vulnerability. 16 It presents a range of symptoms, including hoarseness (the leading symptom), whose degree is reliant on the position of the paralyzed vocal cord, as well as feeble voice, coughing, and aspiration and swallowing issues. 17 18 Breathing issues are also common in UVCP due to shrunk airways. Regarding UVCP treatment, voice therapy, RLN reinnervation, Teflon injections, lipoinjections, as well as thyroplasty, are quite effective. 19 20 21 22

### Etiological Review of Vocal Cord Paralysis

**Figure 2:**
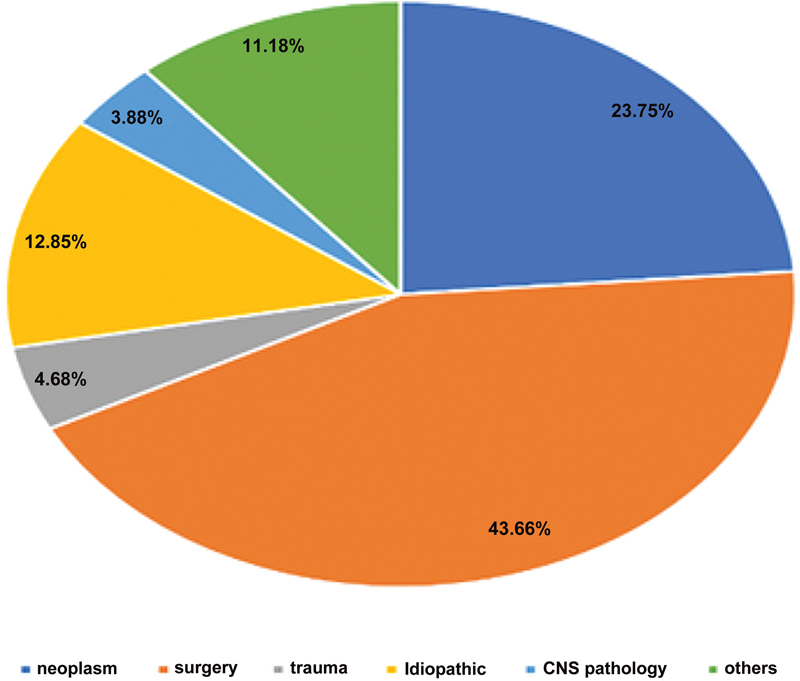
Pie chart illustrating the mean rates of the etiologies associated with vocal cord paralysis.

Previous research has declared vocal cord immobility a disorder with an extensive range of etiologies. 17 The etiology of subject VCP generally varies according to the laterality pattern of the condition ( Figure 2 ).

Research on the etiology of VCP published from 1972 to 2012 mentions surgery as the most prominent cause of VCP, at a mean rate of 43.66% of the cases ( Table 2 ). The most common surgery that causes VCP is thyroidectomy, especially in female patients, due to their higher rate of subclinical thyroid dysfunction (STD). 23 Neoplastic diseases or malignancies are reported as the second most common etiology (at a mean rate of 23.75%) following surgery; approximately half of the neoplastic cases are associated with lung cancer, mostly in male patients, probably due to smoking habits. 4 24

**Table 2:** Meta-analysis of etiologies related to vocal cord paralysis

Total of patients	Neoplasm	Surgery	Trauma	Idiopathic	Central nervous system pathology	
**90**	29%	24%	8%	13%	5%	Srirompotong et al. 50 (2001)
**202**	8%	76%	1%	6%	6%	Neel et al. 10 (1972)
**938**	17.8%	55.6%	3.2%	13.2%	1.9%	Spataro et al. 51 (2014)
**797**	4.2%	50.9%	7.6%	16.2%	3%	Takano et al. 26 (2012)
**84**	40.5%	34.5%	8.3%	10.7%	2.4%	Terris et al. 47 (1992)
**121**	43%	21%	0	18%	5%	Barondess et al. 52 (1986)

**Figure 3:**
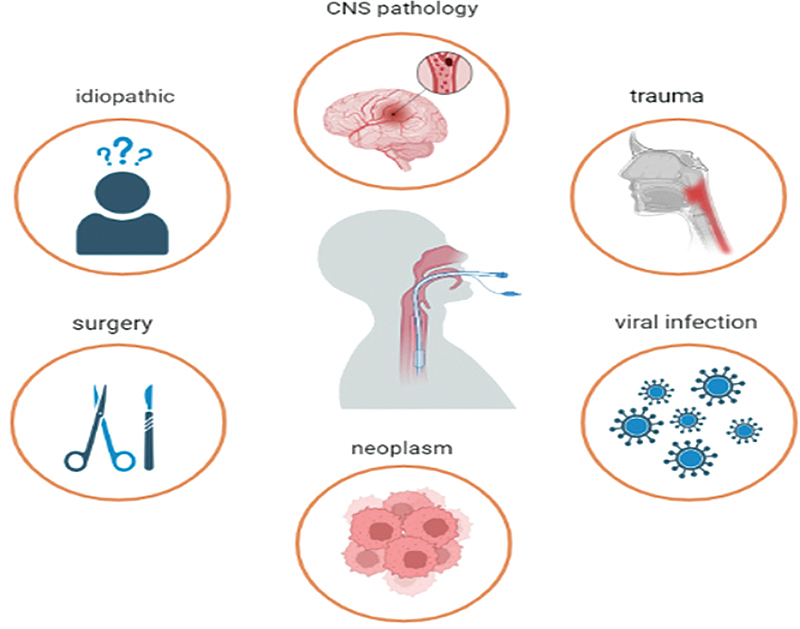
Primary causes of vocal cord paralysis.

One more vital etiology is trauma, at a mean rate of 4.68%. Most congenital cases are idiopathic (mean rate: 12.85%) or associated with central nervous system (CNS) pathologies (mean rate: 3.88%), with links to childbirth trauma, neonatal hypoxia, prenatal anoxia, and Arnold-Chiari malformation. 25 As the word idiopathic indicates, the primary reason for the ailment is unknown, but complications of infections are highly suspected. Due to advancements in diagnostic techniques such as magnetic resonance imaging (MRI), endoscopy, and computed tomography (CT), the rate of idiopathic VCP is rather low. 26 Of all etiologies, the one with the lowest rate, that is, the rarest, is VCP caused by neurological disorders such as Kennedy's disease, vagal mononeuritis, Wallenberg syndrome, and myasthenia gravis. 15 27 Moreover, injury during intubation, viral infection, and ulceration can also cause VCP ( Figure 3 ).

### Vocal Cord Paralysis after Tracheal Intubation

The medical procedure of passing a tube into the trachea through the mouth or nose is termed *endotracheal intubation* ; it is one of the most common practices in general anesthesia to secure the airways and attain first-pass success (FPS) in cases of emergency. Albeit the importance and long establishment of endotracheal intubation in the medical field, it also has some adverse effects on the convalescent patients that range from mild to severe symptoms. During endotracheal intubation, the teeth, esophagus, pharynx, and the trachea, along the intubation pathway, might sustain injury. 28 Sore throat, aspiration and aphonia are common. Symptoms of hoarseness that last for 3 to 4 days after intubation are especially visible in 71% of patients after general anesthesia. On the other hand, hematoma, granuloma, voice loss and tracheal stenosis directly after endotracheal intubation due to injury are considered quite disturbing, because they may result in extended immobility of the larynx. 29 Therefore, otolaryngologists must take this etiology more into consideration.

**Figure 4:**
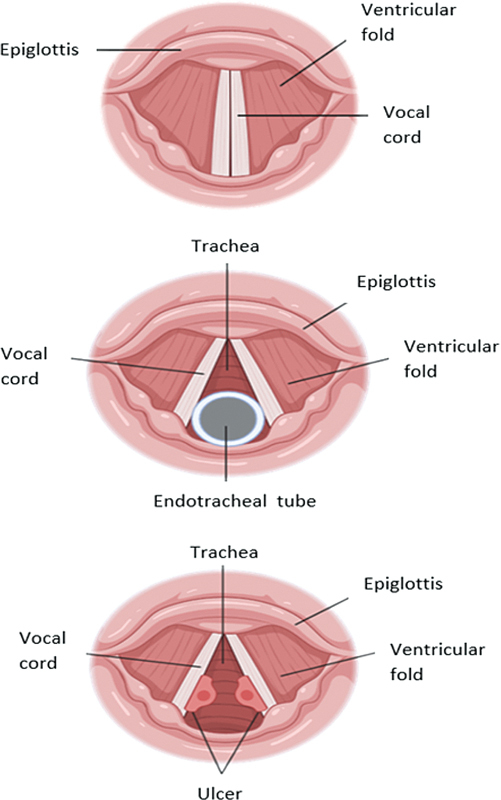
Occurrence of ulcer on the vocal cords due to endotracheal intubation.

Postendotracheal intubation VCP is a relatively rare disorder, but it is a concern for physicians carrying out this procedure. This etiology is responsible for UCVP and BVCP. Endotracheal intubation directly injures the most vulnerable part of the RLN, approximately 6 mm to 8 mm below the cartilaginous glottis. 30 It causes vocal cord immobility that is with comparatively more chances of unilateral by paralyzing the thyroarytenoid muscles and the lateral cricoarytenoid muscles of the RLN. 31 Many demographic factors, such as age and gender, comorbid conditions such as hypertension and diabetes, Body Mass Index (BMI), cuff pressure, operative time, and mishandling of the apparatus have all been reported as risk factors for VCP, since they injure the vocal cords or damage the laryngeal nerve. In total, 68.4% of the patients are reported 32 to recover from post intubation paralysis. All of these risk factors will now be reviewed herein in detail, as the diagnosis of the primary cause of VCP is crucial for the management and treatment of symptoms that appear after endotracheal intubation ( Figure 4 ).

**Age:** Intubation is deemed a safe procedure, but age is considered a major risk factor associated with postendotracheal intubation complications. Individuals aged > 40 years are more likely to present postoperative VCP compared to patients younger than 20 years of age, for whom there have been no reports of postoperative VCP. Dental damage and physiological changes in the joints of the head and the neck occur in elderly individuals; consequently, the tissues of the laryngeal system degenerate with age. Accordingly with the increase in age, the susceptibility to acute swelling or inflammation increases the chances of developing microcirculatory insufficiency due to mechanical injury and cuff pressure. On the contrary, individuals younger than 20 years of age are considered to have a laryngeal system less vulnerable to injury while under endotracheal intubation. This information supports the data reported by Merati et al., 33 who stated that 53.4 years is the mean age associated with a high risk of vocal cord immobility.

**Hypertension:** The risk of postintubation VCP is two times higher in patients with hypertension. In comparison to normotensive patients, hypertensive individuals may have an enhanced hemodynamic response to the induction of anesthesia due to increased sympathetic nervous system activity. 34 Atherosclerotic alterations in the artery vasculature and laryngeal nerve microcirculatory insufficiency are linked to hypertension; that is why in individuals with hypertension, airway tissues may be more susceptible to pressure and mechanical harm from endotracheal intubation. 35

**Diabetes mellitus:** Individuals with diabetes mellitus are more prone VCP as compared with non-diabetic subjects. This may be due to peripheral neuropathy, which is one of the primary complications of diabetes mellitus. 36 The RLN and its branches already present anomalies in diabetic patients; therefore, further neuropathy during intubation will exacerbate the situation enough to cause VCP.

**Cuff pressure, duration of intubation and operative time:** Due to its placement in the larynx, prolonged exposure to the tracheal tube is highly associated with VCP. The pressure exerted by the tracheal cuff during endotracheal intubation causes degeneration of the RLN and its peripheral branches located in the larynx due to the insufficient microcirculatory supply to them. 37 This ultimately leads to RLN paralysis. Cuff pressure can also directly cause ischemic damage or tracheal ulceration. The pressure inside the cuff must not overpass the perfusion pressure of the tracheal mucosa, which is reported to be of approximately 40 cmH _2_ O (29.4 mmHg), 38 and the literature reports that cuff pressures ranging from 19 cmH _2_ O to 40 cmH _2_ O prevent vocal complications. 39 The leading reason for increased intracuff pressure is the quick diffusion into the cuff of the nitrous oxide 40 that is used as a moderate anesthetic in most medical procedures. Using cuffs with low pressure and higher volume is a far better approach than using high pressure and low volume. Additionally, the presence of the tracheal tube for longer periods during intubation may cause physiological alterations due to ulceration, inflammation, erythema and formation of granuloma in the larynx, which might be the primary cause of VCP in most cases. 37 In individuals younger than 20 years of age, the surgery usually takes fewer than three hours; on the other hand, in individuals older than 50 years of age, it usually takes 5 to 6 hours. Kikura et al. 41 reported that short operative time is the primary reason behind the lack of cases of postoperative VCP in people younger than 20 years of age.

**Position of the laryngoscope and malpractice:** The consequences of medical malpractice during endotracheal intubation might be disastrous. The most typical issue regarding the complications of intubation is laryngeal damage, which may cause several illnesses, such as edema and laryngeal inflammation. 42 Moreover, the position and handling of the laryngoscope and endotracheal tube also physiologically affect the larynx and the vocal cords. During intubation, the laryngoscope if fixed on the right side by holding it in the left hand while the tracheal tube is introduced from the right side via holding it in right hand. In this way, the tracheal tube comes directly into contact with the left vocal cord. This is why cases of left-sided VCP are reported twice as much as those of right-sided VCP. 16 43

**Tracheal tube size:** Tracheal tube and cuff size play role a minor risk factor for post tracheal intubation vocal cord paralysis as the larger sized apparatus exert more pressure and enhances the chances of trauma or injury that ultimately leads to VCP. 44 Most of cases of VCP were reported in situation where 8.0mm size of tracheal tube was used. 37 Stout et al. ^44^ used tracheal tube of 7.5 mm and 8.0 mm in male patients; on the other hand, 7.0- and 7.5-mm tracheal tubes were tested. The result did not explicit any reasonable differences.

**Body Mass Index:** A few studies 45 have stated that BMI might be a risk factor associated with postintubation VCP. 45 Arytenoid cartilage and muscles are comparatively weaker in patients with lower BMI, which increases their chances of developing arytenoid dislocation. Vocal fold immobility can occasionally be caused by arytenoid cartilage dislocation, which is seldom recognized. 46

## Final Comments

Depending on the laterality, there are several different etiologies for VCP. Surgeries, cancer, trauma or infections are the main causes of this disorder, with fewer cases attributed to endotracheal intubation. During intubation, damage to the RLN specifically causes UVCP, but 95% of the cases of BVCP are due to scarring in the posterior commissure. Certain demographic factors, such as age, comorbidities (including diabetes mellitus and hypertension), anthropometric factors, such as the BMI, intubation duration, handling of the apparatus, operative time, and tracheal tube sizes may also cause VCP. Preventive measures should be prioritized rather than opting for the treatment of this fatal condition. The proper selection of tube size and cuff pressure, as well as shorter operative times, will help avoid such severe consequences. Additionally, intubation must be done carefully performed in elderly patients or those with lower BMI, as they are more prone to this condition. In conclusion, the risk factors herein reported will help physicians customize the procedure of intubation according to patients and simplify the choice to perform or not perform intubation.
